# 2A-DUB/Mysm1 Regulates Epidermal Development in Part by Suppressing p53-Mediated Programs

**DOI:** 10.3390/ijms19030687

**Published:** 2018-02-28

**Authors:** Christina Wilms, Ioanna Krikki, Adelheid Hainzl, Sonja Kilo, Marius Alupei, Evgenia Makrantonaki, Maximilian Wagner, Carsten M. Kroeger, Titus Josef Brinker, Martina Gatzka

**Affiliations:** 1 Department of Dermatology and Allergic Diseases, University of Ulm, 89081 Ulm, Germany; christinawilms@gmx.net (C.W.); ioanna.krikki@uni-ulm.de (I.K.); adelheid.hainzl@uni-ulm.de (A.H.); marius.alupei@uni-ulm.de (M.A.); evgenia.makrantonaki@uni-ulm.de (E.M.); maximilian-1.wagner@uni-ulm.de (M.W.); carsten.kroeger@uni-ulm.de (C.M.K.); 2 Institute and Out-Patient Clinic of Occupational, Social, and Environmental Medicine, Friedrich-Alexander University, 91054 Erlangen-Nürnberg, Germany; sonja.kilo@fau.de; 3 Department of Dermatology, University Hospital Heidelberg, 69120 Heidelberg, Germany; titus.brinker@nct-heidelberg.de; 4 National Center for Tumor Diseases (NCT), 69120 Heidelberg, Germany

**Keywords:** apoptosis, DUB, epidermal barrier, epidermal stem cell, epigenetics, histone modification, Mysm1, p53, p63, skin

## Abstract

Development and homeostasis of the epidermis are governed by a complex network of sequence-specific transcription factors and epigenetic modifiers cooperatively regulating the subtle balance of progenitor cell self-renewal and terminal differentiation. To investigate the role of histone H2A deubiquitinase 2A-DUB/Mysm1 in the skin, we systematically analyzed expression, developmental functions, and potential interactions of this epigenetic regulator using Mysm1-deficient mice and skin-derived epidermal cells. Morphologically, skin of newborn and young adult Mysm1-deficient mice was atrophic with reduced thickness and cellularity of epidermis, dermis, and subcutis, in context with altered barrier function. Skin atrophy correlated with reduced proliferation rates in Mysm1^−/−^ epidermis and hair follicles, and increased apoptosis compared with wild-type controls, along with increases in DNA-damage marker γH2AX. In accordance with diminished α6-Integrin^high+^CD34^+^ epidermal stem cells, reduced colony formation of Mysm1^−/−^ epidermal progenitors was detectable in vitro. On the molecular level, we identified p53 as potential mediator of the defective Mysm1-deficient epidermal compartment, resulting in increased pro-apoptotic and anti-proliferative gene expression. In Mysm1^−/−^p53^−/−^ double-deficient mice, significant recovery of skin atrophy was observed. Functional properties of Mysm1^−/−^ developing epidermis were assessed by quantifying the transepidermal water loss. In summary, this investigation uncovers a role for 2A-DUB/Mysm1 in suppression of p53-mediated inhibitory programs during epidermal development.

## 1. Introduction

Tissues with high cell turnover, such as the hematopoietic system, the skin, and the intestines, require an elaborate cooperation of chromatin-remodeling and histone-modifying enzymes with sequence-specific transcription factors (TF) and tumor suppressor (TS) genes, to orchestrate the coordinated differentiation and maintenance of stem cells (SC), and to ensure the life-long supply with functional cells [1]. Consequently, in the skin, essentially similar epigenetic enzymes and mechanisms are involved in the control of epidermal development and maintenance, as well as in terminal keratinocyte differentiation, compared with hematopoiesis (reviewed in [2,3,4]). Accordingly, the transcriptional balance of the “epidermal differentiation complex” (EDC), encoding for genes involved in epidermal maturation, such as *loricrin*, *involucrin*, and *filaggrin*, vs epithelial stemness genes, is determined by an intricate interplay of general ATP-dependent chromatin-remodeling enzymes (including Brg1, Satb1, Mi-2β), DNA methyl transferases (Dnmt1), polycomb group (PcG) factors, and histone deacetylases (Hdac1/2), that collaborate with epithelia-related master TF, such as p63, Klf4, Sox9, and others (reviewed in [5,6,7,8]). A major developmental switch involves the interaction of gatekeeper p63, a homologue of TS p53, with Satb1, Brg1, and Klf4—with dual function during embryonic epidermal stratification [9] and during postnatal keratinocyte terminal differentiation [10]. Conversely, polycomb repressive complex (PRC) factors, such as Ezh2 [11] and Bmi1 [12], promote epidermal self-renewal through repression of genes mediating cell cycle inhibition, as well as keratinocyte terminal differentiation. However, the exact mechanisms of the interactions between different epigenetic factors and modifications and sequence-specific TF at different stages of epidermal development and homeostasis are still only incompletely understood. 

Several H2A deubiquitinating enzymes (“DUBs”) counteract the role of PcG proteins in transcriptional repression through H2A deubiquitination (reviewed in [13]). Among these DUBs, MYSM1/Kiaa1915 (Myb-like SWIRM and MPN domain containing1, also 2A-DUB) was first functionally analyzed in prostate cancer cells, where it was shown to bind to double-stranded DNA and activate transcription of androgen receptor (AR) regulated genes by deubiquitinating H2A lysine 119 (H2A-K119ubi) [14]. Mechanistically, MYSM1 regulated transcription in a co-regulatory complex with p/CAF (histone acetyltransferase p300/CBP-associated factor) by coordinating histone acetylation and deubiquitination and destabilizing association of linker histone H1 with nucleosomes [14]. In Mysm1-deficient mouse models, subsequently critical roles of this DUB were uncovered in hematopoietic stem cell (HSC) maintenance and differentiation, in lymphoid cells, and in other cells of the immune system and the bone marrow [15,16]. In support of an interplay between Mysm1 and the p53-axis, defective hematopoiesis and morphological anomalies of Mysm1-deficient mice were functionally rescued in a Mysm1^−/−^p53^−/−^ double-deficient mouse model [17,18]. Changes in Mysm1-deficient lymphoid-primed multipotent progenitors (LMPP) mainly depended on p53 target gene *Puma* [19]. Two recent genetic screens have also implicated Mysm1 in murine skin phenotypes [20,21], presenting initial evidence that Mysm1 may be required for the normal patterning of hair follicles and sebaceous glands in the tail epidermis as well as for skin pigmentation. 

Because our preliminary characterization of Mysm1-deficient mice indicated that increased apoptosis and altered stem cell maintenance may be common denominators in several Mysm1^−/−^ tissues, including the hematopoietic system and skin, we here investigated the skin phenotype induced by Mysm1 loss in more detail. Apart from morphological changes in the Mysm1-deficient epidermis, novel hints for an interplay of Mysm1 with the Arf/p53-axis in the skin and potential roles of Mysm1 in epidermal stem cells (ESC) could be uncovered. 

## 2. Results

### 2.1. Mysm1 Expression in Murine Skin Declines with Age

**Figure 1:**
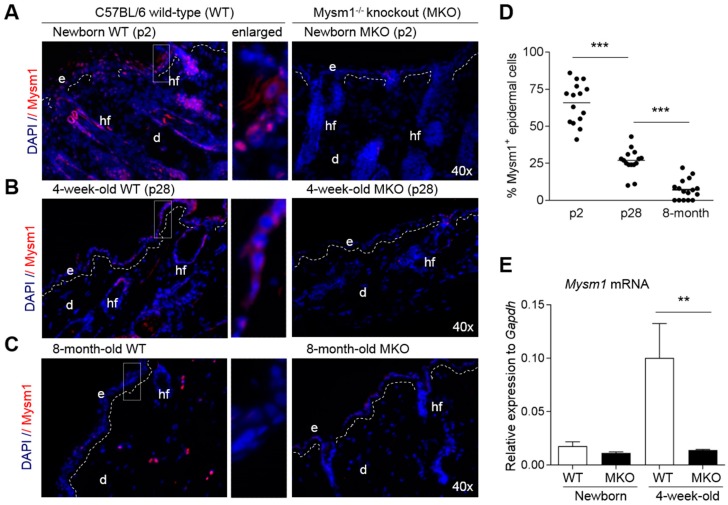
Mysm1 is expressed in murine skin. Analysis of Mysm1 expression by immunofluorescent staining in cryosections of C57BL/6 mice of 3 different age groups compared with Mysm1-deficient controls and by qPCR. (**A**) Newborn mice (NB); (**B**) 4-week-old mice; (**C**) 7–8-month-old mice. (Mysm1 in red, DAPI-stained nuclei in blue, *n* > 3 per age group, representative images; dotted white lines separate e: epidermis and d: dermis, hf: hair follicles); (**D**) quantification of Mysm1-positive cells in the wild-type epidermis in 15 high-power fields (*n* > 3 each age group); (**E**) *Mysm1* mRNA expression in skin cells isolated from either newborn or 4-week-old mice relative to *Gapdh*.

To follow up on our previous data revealing critical roles of Mysm1 in HSC, immune cells, and in melanocyte specification [16,22], we set out to determine Mysm1 expression in the skin compartment of different age groups of wild-type C57BL/6 mice using Mysm1^tm1a^ (MKO) mice as controls. In accordance with a potential role of Mysm1 in skin development, Mysm1 protein expression was highest in newborn mice (p1–3) (Figure 1A)—with successive decline upon aging (Figure 1A–D). In 4-week-old mice, still substantial Mysm1 protein amounts were detectable in epidermis and dermis, whereas 7–8 months old mice showed only minimal Mysm1 expression in all three skin layers (Figure 1B,C). In skin samples from 8-week-old mice, intermediate Mysm1 protein levels compared with younger and older mice were found (not shown). On the mRNA level, however, *Mysm1* was most abundantly expressed in 4-week-old mice (Figure 1E), potentially reflecting changes in the composition of cells upon isolation from full-thickness skin samples or in Mysm1 protein stability. High Mysm1 protein expression in the basal epidermis and hair follicles—representing epidermal stem cell compartments—prompted us to further investigate the role of Mysm1 in skin morphology and epidermal development using Mysm1-deficient (Mysm1^tm1a^, MKO) mice.

### 2.2. Loss of Mysm1 Causes Skin Atrophy and Reduced Skin Cellularity in Mysm1-Deficient Mice

**Figure 2:**
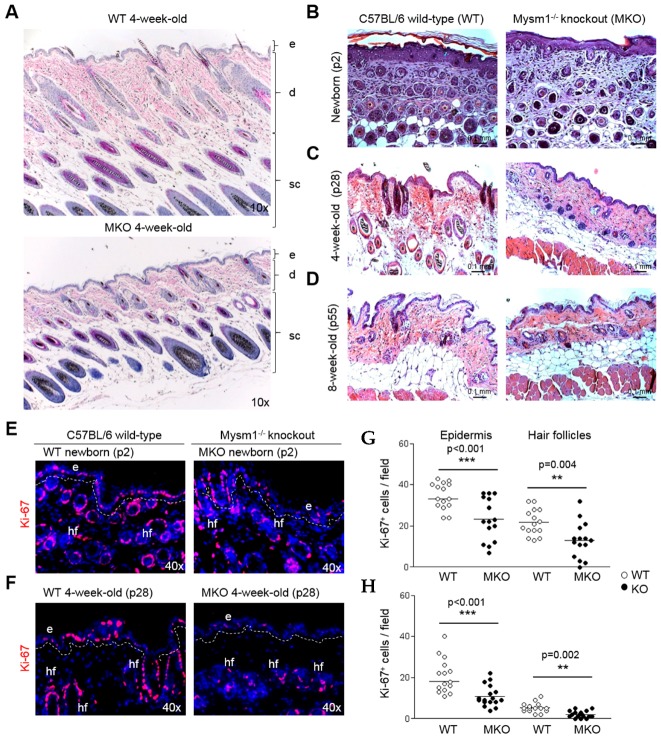
Skin of Mysm1-deficient mice is atrophic. (**A**–**D**) Hematoxylin & Eosin staining of paraffin-embedded skin samples derived from three different age groups of Mysm1-deficient mice and their wild-type littermates, as indicated (e: epidermis, d: dermis, sc: subcutis); (**E**–**H**) IF analysis of proliferation marker Ki-67 in (**E**) newborn and (**F**) 4-week-old Mysm1^−/−^ and wild-type skin, and corresponding statistical summaries (Ki-67 in red, DAPI-stained nuclei in blue, *n* > 4, representative images shown, original magnification 40×, each dot in the graphical summary represents the count of Ki-67^+^ cells in one high power field) in skin of (**G**) newborn and (**H**) 4-week-old mice.

In accordance with the macroscopically thinner and more fragile appearance of the skin of Mysm1-deficient mice of different age groups, H&E staining of full-thickness skin samples (Figure 2A) revealed a reduced overall thickness and cellularity of all three skin layers—epidermis (e), dermis (d), and subcutis (sc)—in newborn MKO mice (p1-3) compared with wild-type (WT) mice at this developmental stage (Figure 2B). In 4-week-old skin, the microscopic differences between WT and MKO were even more pronounced (Figure 2C), and 8-week-old MKO mice still showed moderate skin atrophy (Figure 2D). In Mysm1-deficient dermis, no significant differences in collagen content were detectable in Masson’s trichrome staining—indicating that fibroblast function was not impaired in respective age groups (Supplementary Materials Figure S1). Importantly, blood levels of selected trophic factors impacting skin development, such as insulin-like growth factor 1 (IGF-1), were not significantly altered in 4-week-old Mysm1-deficient mice, as reported in a previous study [23]. 

Differences in skin thickness and cellularity correlated with reduced proliferation rates in epidermis and hair follicles of newborn and 4-week-old Mysm1-deficient mice, compared with wild-type littermates, as indicated by Ki-67 staining in immunofluorescent (IF) analyses (Figure 2E,F). Enumeration of Ki-67^+^-positive cells in these skin sections revealed a highly significant reduction in cellular proliferation in the epidermis and in the hair follicles of newborn (Figure 2G) and 4-week-old Mysm1-deficient mice (Figure 2H). Overall, the analysis of skin structure and composition indicated that Mysm1 potentially has critical functions in epidermal development.

### 2.3. Increased p53-Dependent Apoptosis in Mysm1^−/−^ Skin and Rescue of Skin Atrophy in Mysm1^−/−^p53^−/−^ Mice

**Figure 3:**
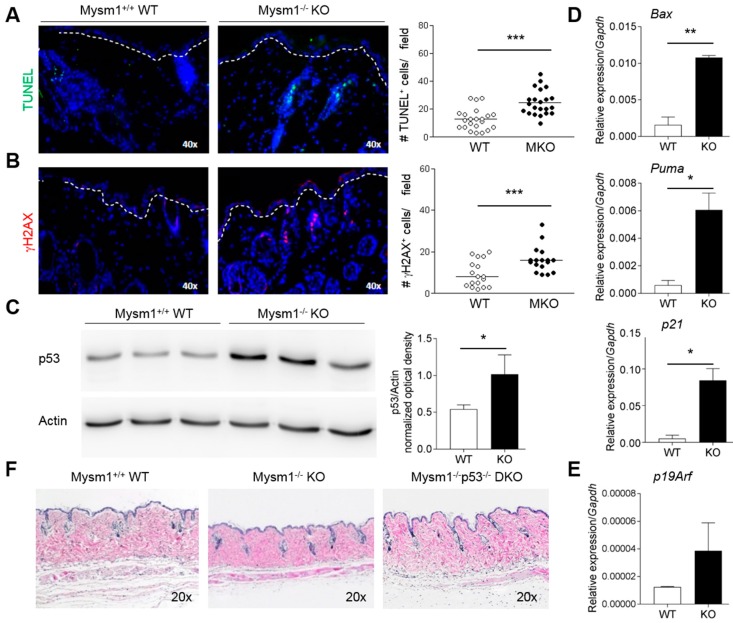
Apoptosis is increased in Mysm1-deficient skin and mediated by p53. (**A**) TUNEL-staining and (**B**) IF analysis of DNA-damage maker γH2AX of cryosections of Mysm1^−/−^ and wild-type 4-week-old skin and statistical summaries (TUNEL-positive cells in green, γH2AX in red, DAPI-stained nuclei in blue, *n* > 4, representative images, original magnification 40×); (**C**) Western blot analysis of p53 protein expression in Mysm1^−/−^ and wild-type skin cell extracts from 4-week-old mice compared to β-actin; (**D**) mRNA expression of p53 target genes and (**E**) of *p19^Arf^* in epidermal samples from 4-week-old Mysm1^−/−^ and wild-type skin by qPCR relative to *Gapdh*; (**F**) H&E staining of skin samples of 8-week-old mice (*n* > 3, original magnification 20×).

To further investigate the causes of skin atrophy of Mysm1-deficient mice, detailed analyses of potential mediators of apoptosis and DNA damage were performed using skin sections, protein, and mRNA. In accord with increased apoptosis observed in Mysm1-deficient hematopoietic cells and other tissues, TUNEL-assays revealed that apoptosis was significantly increased in the skin of 4-week-old Mysm1-deficient mice relative to controls (Figure 3A). Similarly, in line with previous data, increased levels of DNA-damage marker, γH2AX, were detectable in the epidermis and hair follicles of MKO mice (Figure 3B). At the molecular level, p53 protein expression was significantly elevated in whole cell extracts prepared from Mysm1-deficient skin compared with wild-type controls (Figure 3C). Consistently, mRNA expression of selected p53 target genes, such as *Bax*, *Puma*, and *p21^Waf/Cip^*, was significantly increased in MKO vs WT epidermal cells (Figure 3D). Activation of the p53-axis in MKO epidermis could be a consequence of increased mRNA transcription of upstream regulator *p19^Arf^* upon loss of Mysm1 (Figure 3E). Reflecting the causal role of p53 in the changes observed in Mysm1-deficient skin, in Mysm1^−/−^p53^−/−^ double-deficient mice, atrophic skin of Mysm1^−/−^ mice was almost completely restored to normal (Figure 3F).

### 2.4. Mysm1-Deficiency Affects Epidermal Stem Cells

**Figure 4:**
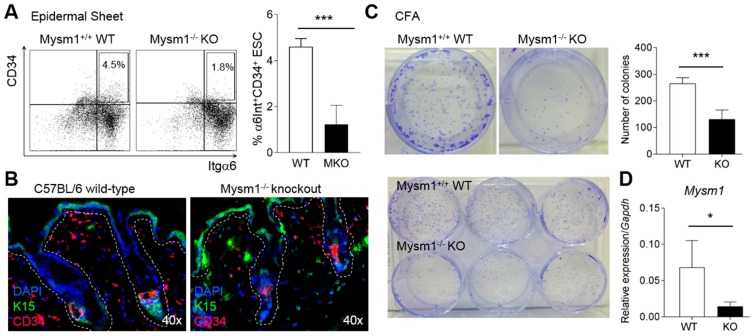
Mysm1-deficiency affects epidermal stem cells. (**A**) FACS-analysis of α6-Integrin (α6Int)^high+^CD34^+^ fractions in epidermal single cell suspensions prepared from 8-week-old Mysm1^−/−^ and wild-type mice (representative dot plots on the left and corresponding bar graphs of mean values on the right, *n* > 6); (**B**) IF analysis of K15^+^CD34^+^ cells in skin cryosections prepared from 4-week-old Mysm1^−/−^ and wild-type mice (K15 in green, CD34 in red, DAPI-stained nuclei in blue, double-positive cells in yellow, *n* > 3, representative images shown, original magnification 40×); (**C**) Colony formation assay (CFA) under keratinocyte differentiation conditions with 4000 epidermal cells per well prepared from 8-week-old Mysm1^−/−^ mice and their wild-type littermates (images of representative wells on the right and statistical summary on the left, *n* > 4, 3 independent experiments); (**D**) *Mysm1* mRNA expression in colonies from either wild-type or Mysm1^−/−^ progenitors relative to *Gapdh*.

Because epidermal stem cells (ESC) residing in the interfollicular epidermis and hair follicle bulges are major contributors to epidermal barrier formation during development, as well as to epidermal homeostasis and wound repair (3), the stem cell composition of Mysm1-deficient epidermis was analyzed in more detail. In fluorescence-activated cell sorting (FACS) analyses of single cell suspensions prepared from epidermal layers of telogen-phase skin from 8-week-old mice (p55), significantly reduced fractions of α6-Integrin (α6Int)^high+^CD34^+^ ESC were detectable in Mysm1-deficient compared with wild-type skin (Figure 4A). Similarly, the fraction of K15^+^CD34^+^ cells in the epidermis and hair follicles of Mysm1-deficient skin samples was reduced in IF analyses of 4-week-old mice relative to age-matched controls (Figure 4B). 

To further analyze the functional contribution of Mysm1 to ESC activity and differentiation, colony formation assays (CFA) were performed under keratinocyte differentiation conditions. Indicative of a role of Mysm1 in ESC specification or differentiation, and in accord with diminished α6Int^high+^CD34^+^ ESC fractions, Mysm1^−/−^ epidermal cells gave rise to reduced colony numbers and colony size in vitro (Figure 4C)—resulting from either reduced precursor numbers or reduced colony formation activity and cell survival. As expected, *Mysm1* mRNA expression was reduced in Mysm1-deficient colonies (Figure 4D). Because mRNA expression of pro-apoptotic genes was not increased in colonies formed by Mysm1^−/−^ epidermal progenitors, we concluded that mainly precursor frequencies of initiating cells were affected by Mysm1 loss.

### 2.5. Defective Epidermal Barrier Formation in Mysm1-Deficient Mice

**Figure 5:**
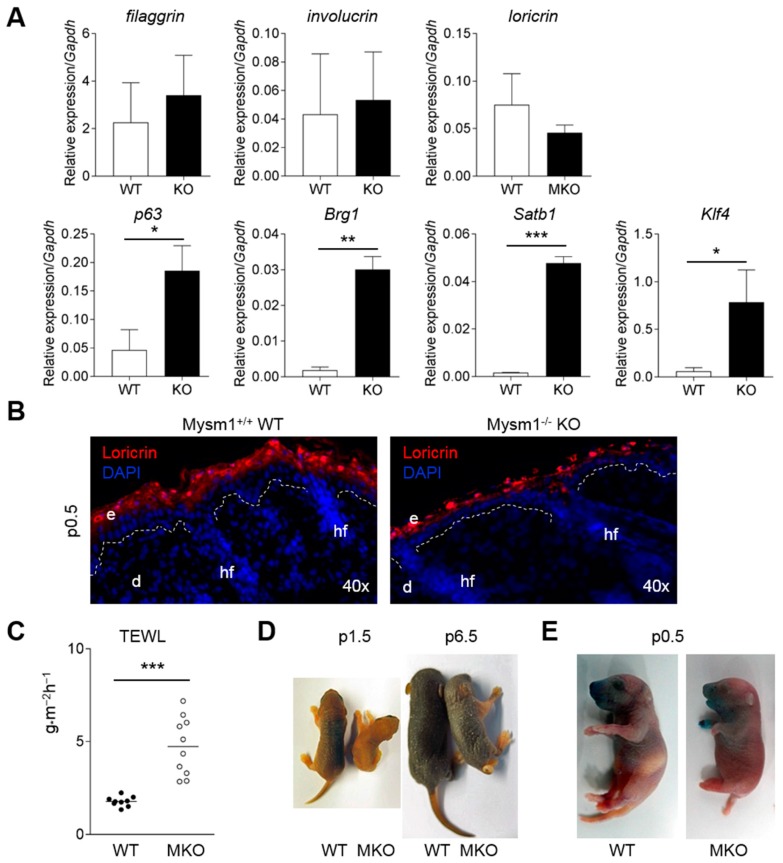
Functionally defective epidermal barrier in newborn Mysm1-deficient mice. (**A**) mRNA analyses of *EDC* genes in newborn (*upper panel*) and transcriptional regulators from 4-week-old mice (*lower panel*) derived epidermal samples relative to *Gapdh* by qPCR; (**B**) IF analysis of Loricrin (red) in skin of newborn WT or Mysm1^−/−^ mice (*n* > 3, representative images, original magnification 40×); (**C**) Quantification of transepidermal water loss (TEWL) on dorsal skin of newborn Mysm1-deficient (MKO) and wild-type (WT) mice (*n* > 3 per genotype, 2 independent measurements per mouse); (**D**) Skin wrinkling of postnatal MKO mice relative to WT controls; (**E**) Toluidine blue dye penetration assay performed with newborn (p1) MKO mice in comparison to WT littermates.

To further analyze the function of Mysm1 in epidermal differentiation, changes in the expression levels of genes of the epidermal differentiation complex (EDC), as well as of TF involved in epidermal barrier formation, were determined in Mysm1-deficient cells from newborn (p0–3) or young adult epidermis (p28–32) compared with wild-type controls. Whereas mRNA expression of *involucrin* and *filaggrin* was not significantly altered in Mysm1-deficient epidermis of newborn mice, *loricrin* mRNA levels were reduced in several Mysm1-deficient mice compared with wild-type littermates (Figure 5A, *upper panel*). In addition, changes in the mRNA expression of TF, such as *p63* and *Klf4*, as well as of chromatin-remodelers, *Satb1* and *Bgr1*, were detectable in 4-week-old Mysm1-deficient epidermis relative to controls (Figure 5A, *lower panel*). At the protein level, Loricrin expression was less abundant in Mysm1^−/−^ epidermis of newborn mice (Figure 5B) and at day E17.5 (Supplementary Materials Figure S2) compared with wild-type littermates in IF analyses. 

Functionally, defects in skin barrier were assessed by quantifying the transepidermal water loss (TEWL) on dorsal skin of live newborn mice (p1–2). Mysm1-deficient mice had significantly increased TEWL compared with their wild-type littermates (Figure 5B); reflecting potential changes in epidermal barrier formation at this age group. Changes in TEWL correlated with visible increased wrinkling (Figure 5C) and an increased perinatal lethality of Mysm1-deficient mice. In addition, mild epidermal barrier defects of newborn (p1) were visualized by Toluidine blue dye penetration (Figure 5D). In context with our data on altered Mysm1^−/−^ skin structure, this investigation of Mysm1 functions in the epidermis identifies novel contributions of this histone modifier to epidermal development and keratinocyte differentiation. Mechanistically, Mysm1 appears to be involved in the suppression of p53-mediated anti-proliferative and pro-apoptotic programs in epidermal cells, as well as in the regulation of transcription networks governing epidermal development and maintenance. 

## 3. Discussion

Based on recent analyses of hematopoietic and immune cell development in Mysm1-deficient mice, two main functions have been attributed to the enzyme 2A-DUB/Mysm1: (1) the transcriptional regulation of target genes involved in development and function of blood cells, such as *Gfi1* or *Ebf2*, as well as (2) functions in suppression of p53-regulated apoptotic programs via target gene regulation [14,15,16,17,18,19]. Consequently, Mysm1^−/−^p53^−/−^ double-mutant mice presented with an overall rescue of the visible morphological deformations and defective hematopoiesis caused by Mysm1-deficiency. In addition, non-canonical functions of Mysm1 in the cytoplasm have been proposed, such as the inactivation of TRAF3 and TRAF6 in innate immune cells during Toll-like receptor (TRL) signaling [24]. Moreover, analyses of human tumor cells, specifically prostate cancer and melanoma cells, revealed that regulation of global H2A deubiquitination by MYSM1 may lead to an overall enhancement of proliferative gene signatures, including androgen-receptor (AR) regulated gene expression [14] and hepatocyte-growth factor (HGF)/c-Met regulated gene expression [25]. However, although the DUB likely affects proliferative tissues in general, Mysm1 functions in other tissues, such as the skin, so far had not been addressed in greater detail. Apart from two genetic screens showing changes in hair follicle patterning in Mysm1-deficient mice [20,21], we here present the first functional data on the role of Mysm1 in epidermal development and function and on potential interactions with p53- und p63-networks in the skin. 

In our investigation of the Mysm1-deficient epidermal compartment, we confirmed two functional mechanisms of Mysm1, because first, p53-mediated apoptosis was increased in Mysm1-deficient skin and epidermal cells, and moreover, expression of certain key transcriptional regulators of keratinocyte specification were altered. In line with a previous report on Mysm1-binding to the *p19^ARF^*-promoter in thymocytes [17], an upstream regulator of TS p53, in Mysm1^−/−^ epidermal cells as well, mRNA expression of *p19^ARF^* was moderately increased along with increases in p53 levels and in apoptosis. Furthermore, reflecting the overall morphological rescue and functional interaction with the p53-axis, the skin atrophy caused by Mysm1-deficiency was largely reversed in young Mysm1^−/−^p53^−/−^ double-deficient mice. Along with increased apoptosis, α6Int^high+^CD34^+^ ESC fractions were reduced in adult Mysm1-deficient skin. Physiologically, apoptotic programs have key roles in the self-renewal process of squamous epithelia and need to be coordinated with terminal differentiation programs (reviewed in [25]). The PCR1 factor Bmi1 has previously been implicated in epidermal cell survival and proliferation via suppression of the *Ink4a*-*Arf* locus [12]. Comparable to regulatory events in early thymocyte development, Bmi1 and Mysm1, with counteracting effect on H2A-K119ubi, may therefore interact dynamically in *Cdkn2a*-promoter regulation, affecting ESC survival and self-renewal. Physiologically decreasing Mysm1 expression in the skin with age may indicate that the deubiquitinase is mainly involved in development, compared with additional roles of Bmi1 in epidermal homeostasis and aging via p16^Ink4a^ [26,27]. The relatively mild skin phenotype of Mym1^tm1a^ mice, that do not develop any ulcers or desquamation, may in part reflect the hypomorphic gene mutation with remaining low Mysm1 expression [14]. 

Among established regulators of epidermal stratification and differentiation, expression of *p63* was deregulated in Mysm1-deficient epidermis in context with altered expression of *Brg1*, *Satb1*, and *Klf4*. Because the p63-Brg1/Satb1-Klf4-axis is considered to be a vital developmental switch regulating embryonic epidermal stratification and terminal keratinocyte differentiation [9,10], we conclude that increased expression of all factors of this axis in the Mysm1-deficient epidermis may reflect a compensatory mechanism—potentially related to increased p53-mediated programs or increases in reactive oxygen species (ROS) in Mysm1-deficient skin [14,17]. ROS levels and stress-associated signaling via p38-kinases have previously been shown to enhance transcriptional networks in a positive feedback loop, leading to increased epidermal expression of *p63* and its target genes involved in keratinocyte terminal differentiation [28,29]. Alternatively, Mysm1 may influence *p63* transcription by counteracting chromatin changes induced by PRC1 component Bmi1—with context-dependent activatory or inhibitory influence in stem cells [5], [and unpublished meeting communications]. Indicative of an impact of Mysm1 on early epidermal differentiation, potentially through an interaction with the p63-axis, expression of EDC-component *loricrin* was reduced in several newborn Mysm1*^−/−^* mice, along with mild defects in the epidermal barrier and increases in transepidermal water loss in this age group. More detailed analyses of EDC components and transcription factors during MKO embryogenesis may therefore be of relevance, because the expression of *loricrin* physiologically first peaks at E16.5–E17.5 [30]. In support of differential roles of epigenetic regulators during early epidermal development and adult epidermal homeostasis, PcG factor Cbx4, contributing to H2A-K119 mono-ubiquitination in cooperation with Bmi1/Ring1b downstream of p63, was shown to be involved in the regulation of epidermal thickness in adult mice via repression of cell cycle inhibitory genes *p16/p19* and *p57*, along with a repressive effect on premature expression of EDC genes in the developing epidermis, and on non-epidermal lineage (neuronal) genes in epidermal precursors [31]. 

In context with declining Mysm1 expression levels in the murine skin with age, we conclude that Mysm1 is a critical epigenetic regulator of epidermal development and ESC specification through interference with p53-mediated apoptosis and cell cycle inhibition—and potentially modification of p63-regulated programs—whereas ESC maintenance later in adult life may be compensated through yet to be investigated mechanisms. Future research addressing the interaction of Mysm1 with other epigenetic regulators and also with environmental factors, such as UV light, in the skin, will likely provide important new insights into skin biology and diseases.

## 4. Materials and Methods

### 4.1. Mouse Models

Mysm1^tm1a(Komp)Wtsi^ mice (Mysm1^−/−^, MKO) and Mysm1^−/−^p53^−/−^ mice (DKO) have been described previously [17], and were handled in accordance with the guidelines for animal experimentation approved by the Regierungspräsidium Tübingen, Germany (#1162, 08/2014; #1261, 16 June 2016). Skin samples from postnatal (p1-3), 4-week-old (p28), 8-week-old (p55), and 7–8-months-old MKO mice and their wild-type (WT) littermates were used for analyses as indicated (*n* > 3). 

### 4.2. Histology and Immunofluorescent Analyses

Paraffin-embedded or cryo-preserved tissue samples were routinely processed and stained as previously described [17] and analyzed using an Axio Imager microscope (Zeiss, Jena, Germany). Hematoxylin & Eosin (H&E) and Masson’s Trichrome stainings were performed according to established protocols [32]. For immunofluorescent (IF) analyses, specific antibodies against Mysm1 (R72303, Sigma Aldrich/Atlas Antibodies, Taufkirchen, Germany), Ki-67, K15, CD31, CD34, p19^Arf^/Cdkn2a, and γH2AX, as well as secondary antibodies (all either from Abcam, Cambridge, UK, Sigma-Aldrich, or DAKO, Jena, Germany) and antibodies against Loricrin and Involucrin (Biolegend, San Diego, CA, USA), were used as indicated. Isotype IgG served as negative control in all experiments. Nuclei were visualized by 4′,6-Diamidin-2-phenylindol (DAPI) staining. Terminal deoxynucleotidyl transferase dUTP nick end labeling (TUNEL) assays were performed according to the manufacturer’s protocol (In Situ Cell Death Detection Kit, Sigma-Aldrich, Taufkirchen, Germany). Original magnification was 40×, and representative slides of at least 3 independent samples are shown, unless indicated otherwise. 

### 4.3. qPCR

Total RNA was prepared from skin epidermis or epidermal stem cell-derived colonies, reverse transcribed, and analyzed by qRT-PCR (Roche, Basel, Switzerland), as previously described [17]. Expression levels of target genes were calculated by normalization to *Gapdh* mRNA expression. 

### 4.4. Western Blot

Cell extracts were prepared from skin samples using whole cell lysis buffer containing 10% glycerol, 1% Triton-X100, and protease and phosphatase inhibitors (Thermo Scientific, Waltham, MA, USA). Subsequently, 100 µg protein per sample was separated by SDS-PAGE using 10% polyacrylamide gels, transferred onto a nitrocellulose (NC) membrane (GE Healthcare Life Sciences, Freiburg, Germany), and analyzed upon incubation with antibodies against p53 and β-actin (both from Cell Signaling, Danvers, MA, USA), as previously described [33]. 

### 4.5. Colony Formation Assay (CFA)

For primary murine epidermal stem cell colony formation assays, epidermal cells were isolated from back skin of 8–10-week-old mice following established protocols [34]. Subsequently, 4000 epidermal cells were seeded per well of a 6-well plate pre-coated with a mitotically arrested 3T3 fibroblast feeder cell layer, and incubated in DMEM/HAM’s medium (Biochrom, Berlin, Germany) supplemented with Ca^2+^ free FCS, l-glutamine, penicillin/streptomycin, ascorbic acid, epidermal growth factor (EGF), insulin, adenine, cholera toxin, and hydrocortisone at 32 °C for 2–3 weeks [35]. Post incubation, following removal of feeder cells, colonies were stained with 0.5% crystal violet (Sigma-Aldrich, Taufkirchen, Germany) in methanol for 10 min at room temperature, washed, and counted manually.

### 4.6. Transepidermal Water Loss (TEWL)

TEWL measurements were performed in duplicate on dorsal skin of postnatal mice (p1–3) by the open chamber method using a Tewameter^®^ V300 (Courage, Khazaka Electronic, Cologne, Germany). Data was expressed in g∙m^−2 ^h^−1^, and represents the mean ± SEM from at least six independent animals of each genotype.

### 4.7. Statistical Analyses

Statistical significance of the results was tested by one-way analysis of variance (ANOVA) and Fisher’s LSD post hoc test. The results were presented as the mean ± standard deviation (SD). Significance levels were set to * = *p* < 0.05, ** = *p* < 0.01, *** = *p* < 0.001 unless indicated otherwise. 

## Supplementary Materials

Supplementary materials can be found at http://www.mdpi.com/1422-0067/19/3/687/s1.
