# Supplementary material for: 2A-DUB/Mysm1 Regulates Epidermal Development in Part by Suppressing p53-Mediated Programs
Source: Int J Mol Sci. 2018 Feb 28;19(3):687. doi: 10.3390/ijms19030687 (PMC5877548; doi:10.3390/ijms19030687)
Supplement: Supplementary file 1 [file ijms-19-00687-s001.pdf]

Figure S1.

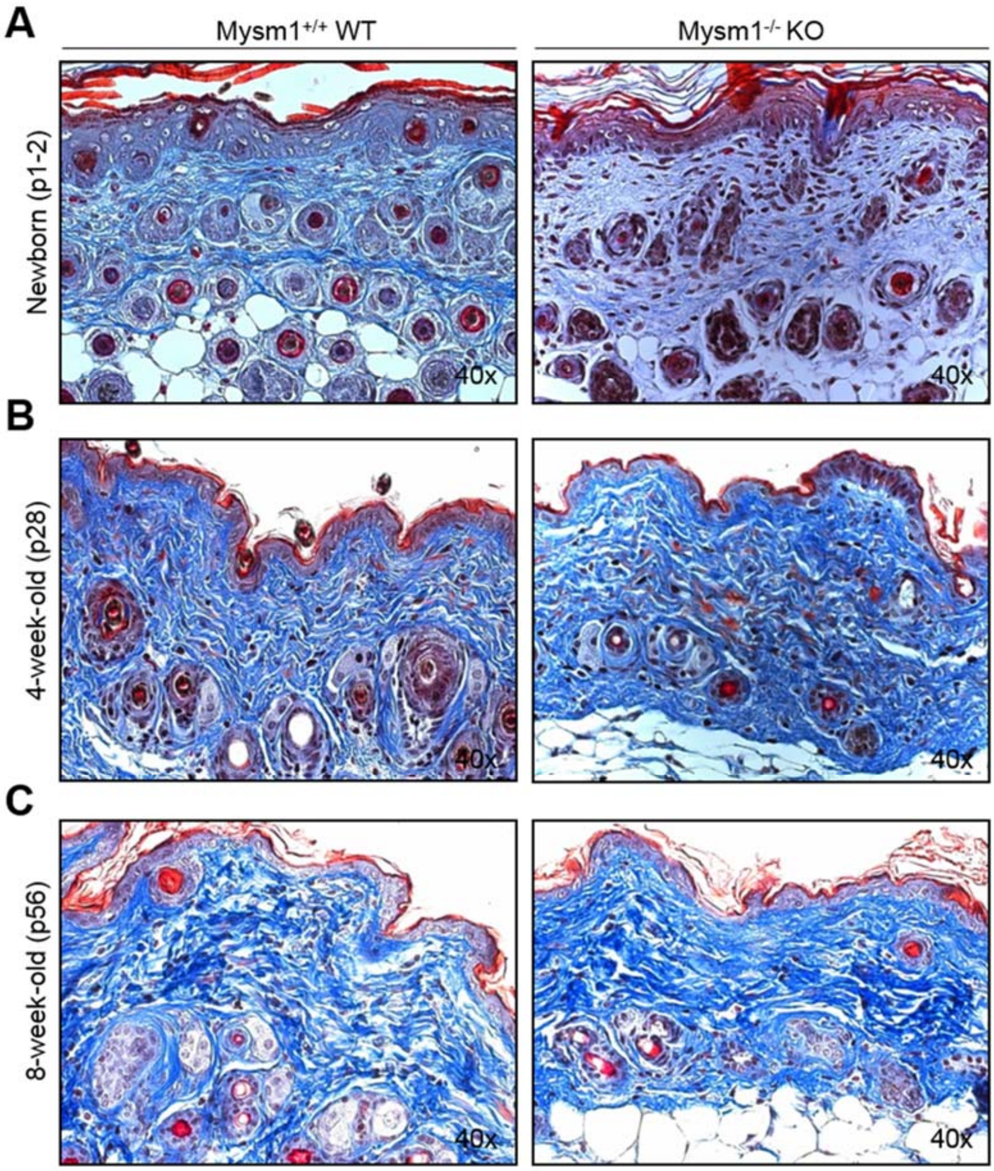

Fig S1: **Collagen deposition in Mysm1-deficient skin**. Masson Trichrome stainings of mouse skin sections of (**A**) newborn, (**B**) 4-week-old and (**C**) 8-week-old Mysm1$^{-/-}$ mice in comparison with wild-type littermates (n>3, representative images, original magnification 40X).

Figure S2.

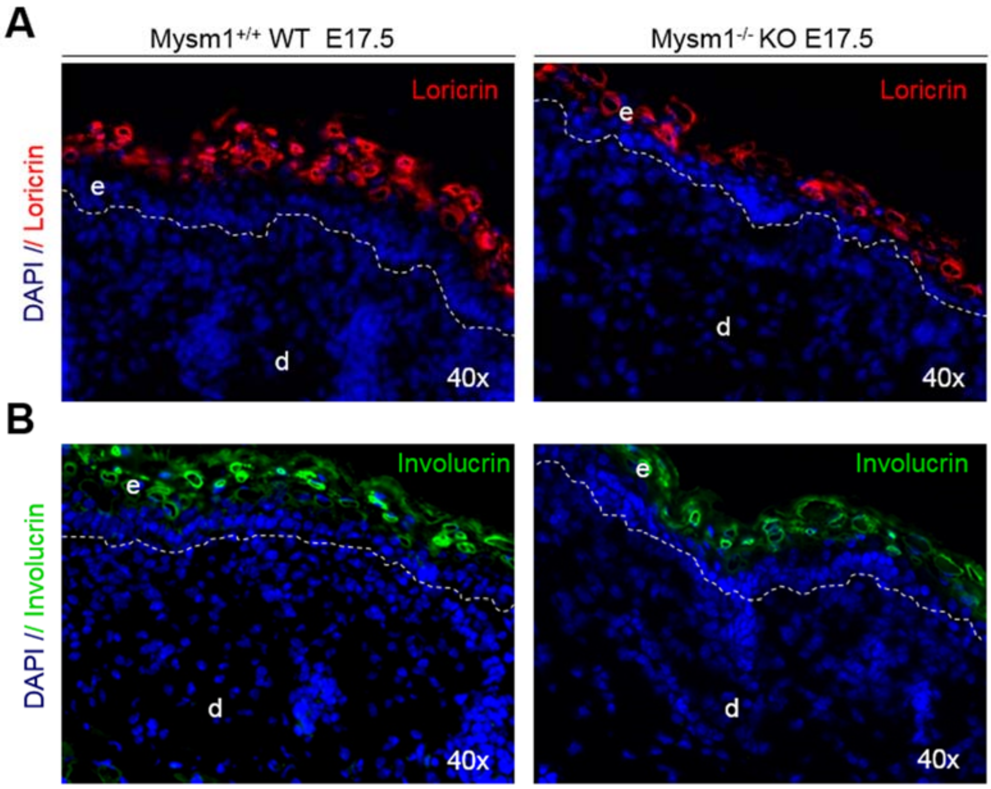

Fig. S2: **Expression of EDC proteins in Mysm1-deficient epidermis during development**. IF analysis of E17.5 skin of Mysm1-deficient in comparison with wild-type mice (n>3, representative images shown, dotted white lines separate e: epidermis and d: dermis, original magnification 40X). (**A**) Loricrin expression (Loricrin red, DAPI-stained nuclei blue). (**B**) Involucrin expression (Involucrin green, DAPI-stained nuclei blue).
